# Modeling Ghanaian cocoa farmers’ decision to use pesticide and frequency of application: the case of Brong Ahafo Region

**DOI:** 10.1186/s40064-016-2779-z

**Published:** 2016-07-19

**Authors:** Elisha Kwaku Denkyirah, Elvis Dartey Okoffo, Derick Taylor Adu, Ahmed Abdul Aziz, Amoako Ofori, Elijah Kofi Denkyirah

**Affiliations:** Department of Agricultural Economics and Agribusiness, University of Ghana, P. O. Box LG 68, Legon, Accra, Ghana; Institute for Environment and Sanitation Studies (IESS), University of Ghana, P. O. Box 209, Legon, Accra, Ghana; Department of Crop Science, University of Ghana, P. O. Box LG 44, Legon, Accra, Ghana; Department of Agricultural Economics and Rural Development, University of Gottingen, Platz der Göttinger Sieben 5, 37073 Göttingen, Germany

**Keywords:** Pesticide, Cocoa farmers, Decision to use pesticide, Frequency of pesticide application, Probit and Tobit regression models, Berekum Municipality, Ghana

## Abstract

Pesticides are a significant component of the modern agricultural technology that has been widely adopted across the globe to control pests, diseases, weeds and other plant pathogens, in an effort to reduce or eliminate yield losses and maintain high product quality. Although pesticides are said to be toxic and exposes farmers to risk due to the hazardous effects of these chemicals, pesticide use among cocoa farmers in Ghana is still high. Furthermore, cocoa farmers do not apply pesticide on their cocoa farms at the recommended frequency of application. In view of this, the study assessed the factors influencing cocoa farmers’ decision to use pesticide and frequency of pesticide application. A total of 240 cocoa farmers from six cocoa growing communities in the Brong Ahafo Region of Ghana were selected for the study using the multi-stage sampling technique. The Probit and Tobit regression models were used to estimate factors influencing farmers’ decision to use pesticide and frequency of pesticide application, respectively. Results of the study revealed that the use of pesticide is still high among farmers in the Region and that cocoa farmers do not follow the Ghana Cocoa Board recommended frequency of pesticide application. In addition, cocoa farmers in the study area were found to be using both Ghana Cocoa Board approved/recommended and unapproved pesticides for cocoa production. Gender, age, educational level, years of farming experience, access to extension service, availability of agrochemical shop and access to credit significantly influenced farmers’ decision to use pesticides. Also, educational level, years of farming experience, membership of farmer based organisation, access to extension service, access to credit and cocoa income significantly influenced frequency of pesticide application. Since access to extension service is one key factor that reduces pesticide use and frequency of application among cocoa farmers, it is recommended that policies by government and non-governmental organisations should be aimed at mobilizing resources towards the expansion of extension education. In addition, extension service should target younger farmers as well as provide information on alternative pest control methods in order to reduce pesticide use among cocoa farmers. Furthermore, extension service/agents should target cocoa farmers with less years of farming experience and encourage cocoa farmers to join farmer based organisations in order to decrease frequency of pesticide application.

## Background

Agriculture plays a significant economic role for many countries in West Africa. Indeed, the importance of agriculture to the growth of the Ghanaian economy cannot be overemphasized in relation to the labour force it attracts. Agriculture is the largest sector of the Ghanaian economy and the highest contributor to Ghana’s GDP, employing about 60 % of the country’s labour force (ISSER 2010). The agricultural sector in Ghana is dominated by tree crops such as cocoa, coffee, oil palm and rubber. Among these tree crops, cocoa is of particular interest for Ghana and for the global chocolate industry (Danso-Abbeam et al. 2014). The cocoa sector represents more than half (70–100 %) of the income for roughly 800,000 smallholder farm families in Ghana, providing food, employment, tax revenue and foreign exchange earnings for the country (Appiah 2004; Anim-Kwapong and Frimpong 2004; Ayenor et al. 2007; Anang 2011; Danso-Abbeam et al. 2014).

Despite the economic importance of cocoa, its production in Ghana is threatened by insect pests and diseases, a situation which has resulted in the decline in cocoa production, with adverse impact on the Ghanaian economy. A significant component of the modern agricultural technology which has been widely adopted by cocoa farmers in Ghana to prevent or control insect pests and diseases in order to reduce or eliminate yield losses in cocoa and to maintain high product quality is pesticide.

However, the use of pesticide in agriculture, and for that matter the cocoa industry in Ghana has raised a lot of concerns about the safety of residues in cocoa beans, soils and water, as well as other potential harm to humans and the environment (e.g. destruction of natural enemies of pest and the development of pest resistance) (Antle and Pingali 1994; Pimentel 2005; Adeogun and Agbongiarhuoyi 2009; Hou and Wu 2010; Adejumo et al. 2014). In most developing countries like Ghana, these consequences have often been severe because farmers do not use approved pesticides, and do not follow recommended frequencies of pesticide application by government agencies for crops. They however misuse, overuse and apply pesticides indiscriminately (Konradsen 2007; Sam et al. 2008), with disregard to safety measures and regulations on chemical use.

This has expose farmers to risk due to hazardous effects of these chemicals. According to Atu (1990), pesticides are toxic and can have serious health hazards on human beings. WHO/UNEP (1990) reported that the use of pesticides is responsible for 3 million acute poisoning and results in about 20,000 deaths of farm workers annually mostly in developing countries. It is also reported that exposure to pesticides have long term effects on thyroid function, cause low sperm count in males, birth defects, increase testicular cancer, reproductive and immune malfunction/problems, endocrine disruptions, dermatitis, behavioural changes, cancers, immunotoxicity, neurobehavioral and developmental disorders (PAN International 2007; Mesnage et al. 2010; Tanner et al. 2011; Cocco et al. 2013; Gill and Garg 2014). Furthermore, Ntow et al. (2006), Pan-Germany (2012) and Gill and Garg (2014) reported on the short term effects such as headaches, body aches, skin or eye irritation, respiratory problems, weakness, dizziness, impaired vision and nausea as a result of pesticide exposure.

Although studies have revealed that pesticide use poses threats to the environment and farmers themselves, and that farmers can improve yields as well as increase profits following adoption of integrated pest management (IPM), integrated plant nutrition systems (IPNS) and other technologies (Pretty 1995), pesticide use is still high among farmers in Ghana, particularly, cocoa farmers. The question that arises is “what are the factors that influence the decision of a farmer to use pesticide”? Another major concern aside the use of pesticide is the frequency of pesticide application. The Ghana Cocoa Board (COCOBOD) recommends that for effective and sustainable control of pests and diseases, cocoa farmers need to apply pesticides on their cocoa farms four times per season (Adu-Acheampong et al. 2007). This notwithstanding, farmers do not apply pesticide on their cocoa farms at the recommended frequency. The question that arises is “what are the factors that influence frequency of application”? Knowing the factors that influence pesticide use and frequency of application would enable stakeholders such as Ghana COCOBOD and the Ministry of Food and Agriculture (MoFA), to identify the specific issues (socio-economic characteristics) that influences cocoa farmer’s pesticide use and frequency of application in order to put up policies (such as Cocoa Disease and Pest Control (CODAPEC) programme and IPM Farmer Field Schools) that would reduce or increase pesticide use and frequency of application if necessary. In view of this, it is imperative to assess the factors that influence cocoa farmers’ decision to use pesticide and frequency of pesticide application.

Although studies have assessed factors influencing farmers’ choice of pesticide or use of pesticide (Adejumo et al. 2014; Anang and Amikuzuno 2015), little information is known in the case of Ghana, particularly, on cocoa farmers. Also, studies which sort to assess frequency of pesticide application (Avicor et al. 2011; Oesterlund et al. 2014; Antwi-Agyakwa et al. 2015), only described the frequency of pesticide application and did not estimate the factors which influence frequency of pesticide application.

One of the major cocoa producing regions in Ghana is the Brong Ahafo Region. In order to control insect pests and diseases and increase cocoa yield, farmers in the region use pesticides extensively. These chemicals are however used improperly or in dangerous combinations with disregard for approved pesticides and recommended frequency of application by Ghana COCOBOD for cocoa production. In view of this, the question that arises is “why do cocoa farmers use approved pesticides in combination with unapproved pesticides and with disregard for the recommended frequency of application”? Unfortunately, there is little documentation on pesticides management by cocoa farmers in the region. This study therefore seeks to analyse the pesticides used by cocoa farmers, frequency of pesticide application, the factors influencing cocoa farmers’ decision to use pesticide and the factors influencing frequency of pesticide application by cocoa farmers in the Berekum Municipality of the Brong Ahafo Region of Ghana.

## Methods

### The study area

The study was carried out in the Berekum Municipality. It lies between latitude 7′15° South and 8′00° North and longitude 2′25° East and 2′50° West. Berekum Municipality lies in the North-western corner of the Brong Ahafo Region of Ghana. The Municipality covers total land area of about 863.3q.km. The Municipality lies within the wet semi-equatorial climate zone which occurs widely in the tropics and it experiences a maxima pattern of rainfall with a mean annual rainfall ranging between 1275 and 1544 mm in May to June (Berekum Municipal Assembly report 2013). Basically the Municipality has the most semi-deciduous forest type of vegetation which covers 80 % of the entire stretch of the land. The population of Berekum Municipality, according to the 2010 Population and Housing Census, is 129,628 representing 5.6 percent of the region’s total population. More than half (57.0 %) of households in the municipality are engaged in agriculture. Most households engaged in agriculture in the municipality (97.6 %) are involved in crop farming (Ghana Statistical Service 2014). Soils in the municipality fall into the ochrosols group which is generally fertile and therefore support the cultivation of cocoyam, maize, cassava, cocoa and plantain.

### Sampling technique and sample size

A total of 240 cocoa farmers were selected for the study using the multi-stage sampling technique. At the first stage, the Brong Ahafo Region of Ghana was purposively selected due to the predominance of cocoa production in the region. At the second stage, the Berekum Municipality was randomly selected out of the several cocoa producing districts in the region. At the third stage, six (6) cocoa growing communities, namely, Koraso, Kutre no. 1 and 2, Senase, Kato, Biadan and Ayimom in the district were randomly sampled. At the fourth stage, a minimum of forty (40) cocoa farmers were selected from each of the six cocoa growing communities. All participants agreed to participate in the research study by signing informed consent forms.

### Instrumentation for data collection

A pre-tested semi-structured questionnaire was developed as an instrument for data collection. The structure of questions in the data collection instrument was a combination of close-ended, open-ended and partially close-ended questions. The survey was conducted from March 2015 to July, 2015.

### Analytical framework

A farmer is assumed to make choices or adopt a particular technology which maximizes his or her utility. The choice a farmer makes to either use or not to use a particular technology is estimated using the discrete choice models. The two models used to estimate farmers’ choice to use a particular technology or not are the logistic regression or logit and probabilistic regression or probit models. The dependent variable of these models takes the form of a dummy variable equal to 1 if a farmer chooses to use a particular technology and 0 if otherwise. The major difference between these two models is the distribution of the error term, ε. For the logit model, the error term is assumed to have the standard logistic distribution while the error term for the probit model is assumed to have the standard normal distribution (Bryan et al. 2009). The probit model was adopted for this study because it has the ability to resolve the problem of heteroscedasticity and also has the ability to constrain the estimated probabilities to lie between 0 and 1 (Asante et al. 2011). Again, economists tend to prefer the normality assumption of the probit model, given that several specification problems are more easily analyzed because of the properties of the normal distribution (Wooldridge 2006). If we assume a dependent variable Y having only two possible outcomes as 1 and 0 and which is influenced by independent variables X, the probit model takes the form:

1 $${\text{Pr(}}Y = 1|X) = \varphi (X^{\prime } \beta )$$

where Pr denotes probability and *φ* denotes the cumulative distribution function of the standard normal distribution. The maximum likelihood analysis is used to estimate the parameters (β). The probit model can further be written as:

2 $$Y = F(\alpha + \beta x_{i} ) = F(z_{i} )$$

where Y denotes the discrete choice variable; F denotes the cumulative probability distribution function; *β* denotes the vector of parameters; *x* denotes the vector of explanatory variables; *z* denotes the Z-score of *βx* for the area under the normal curve.

The probit model can be specified as a linear function of the variables that determine the probability:

3 $$Y = \beta_{0} + \beta_{i} X_{i} + \cdots + \beta_{n} X_{n}$$

Marginal effect is estimated for *Xi*. The marginal effect of *X*_*i*_ is ∂*p*/∂*X*_*i*_ and is computed as:

4 $$\partial p/\partial X_{i} = \frac{\partial p}{\partial Y} \, \frac{\partial Y}{{\partial X_{i} }} = f(Y)\beta_{i}$$

*f* (Y) is the derivative of the cumulative standardized normal distribution and is just the standardized normal distribution itself:

5 $$f(Y) = \frac{1}{{\sqrt {2\pi } }}e^{{ - \frac{1}{2}Z^{2} }}$$

The cumulative standard normal distribution is given as *F* (*Y*) and it gives the probability of the event occurring for any value of Y:

6 $$P_{i} = F(Y)$$

Furthermore, the Tobit regression model was used to estimate the frequency of pesticide application. This is because there is a possibility that not all the farmers may use pesticides. Frequency of pesticide application for such group of farmers who do not use pesticide was captured as zero. The Tobit model is a better choice than the ordinary least square estimates because the ordinary least square presents censoring bias. Also, the Tobit model interprets all the zero observations in the data set as corner solution. This model has been used in many studies (Nkamleu 2004; Holloway et al. 2004; Oladede 2005; Nkamleu et al. 2007; Nkamleu and Tsafack 2007) to estimate farmers’ adoption of technology packages.

$$FPA_{i} = FPA_{i}^{*} \quad {\text{if}}\;FPA_{i}^{*} > 0$$

$$FPA_{i}^{*} = \, 0\quad {\text{if}}\,\,{\text{otherwise}}$$

7 $$FPA_{i}^{*} = xi^{\prime } \beta + ui$$

where $FPA_{i}^{*}$ is the observed response on the frequency of pesticide application. *x* is the vector of independent variables, *β* is a vector of parameters and *ui* is the error term which is randomly distributed.

### Empirical model

The probit model was used for this work to analyse cocoa farmers’ decision to use pesticides on their farms. The probit model is specified for this study as:

8 $${\text{Y }} = \beta_{0} + \beta_{1} X_{1} + \beta_{2} X_{2} + \beta_{3} X_{3} + \beta_{4} X_{4} + \beta_{5} X_{5} + \beta_{6} X_{6} + \beta_{7} X_{7} + \beta_{8} X_{8} + \beta_{9} X_{9} + \varepsilon$$

Y = dependent variable (1 = pesticide use and 0 = otherwise), β_0_ = coefficient of constant term, β_1_–β_9_ = coefficient of the independent variables, X_1_–X_9_ = explanatory variables, ε = error term.

The Tobit model was further used to estimate farmers’ frequency of pesticide application. The empirical model is specified for this study as:

9 $${\text{FPA}} = \beta_{0} + \beta_{1} X_{1} + \beta_{2} X_{2} + \beta_{3} X_{3} + \beta_{4} X_{4} + \beta_{5} X_{5} + \beta_{6} X_{6} + \beta_{7} X_{7} + \beta_{8} X_{8} + \beta_{9} X_{9} + \varepsilon$$

FPA = Frequency of pesticide application, β_0_ = coefficient of constant term, β_1_–β_9_ = coefficient of the independent variables, X_1_–X_9_ = explanatory variables, ε = error term.

The explanatory variables are defined as follows:

X_1_ denotes gender, X_2_ denotes age of farmer, X_3_ denotes educational level, X_4_ denotes years of farming experience, X_5_ denotes access to extension, X_6_ denotes membership of Farmer Based Organisation (FBO), X_7_ denotes availability of agrochemical shop, X_8_ denotes access to credit and X_9_ denotes cocoa income.

### Explanation of variables

Table 1 presents the description, measurements and a-priori expectations of explanatory variables used in the study. In the case of gender, male farmers are expected to use pesticides more often and at a higher application rate than female farmers. Females are said to be more vulnerable to pesticide exposure (Engel et al. 2005; Goldner et al. 2010), which could lead to a decreased use of pesticides among female farmers. With respect to age, younger farmers are more likely to adopt new technologies than older farmers (Alavalapati et al. 1995; Adejumo et al. 2014). Therefore, it is expected that older farmers would use pesticide compared to younger farmers. In regards to availability of agrochemical shop, farmers tend to use technologies readily available to them in order to save time in search of technologies which are not readily available (Anang and Amikuzuno 2015). Therefore, availability of agrochemical shop would positively influence farmers to use pesticide and increase frequency of application. It is noted that credit positively influence pesticide use and frequency of application, because farmers are able to purchase more pesticides regardless of the cost (Abu et al. 2011). Similarly, increase in cocoa income helps farmers to purchase more pesticides regardless of the cost. Farmers who attain higher level of education are less likely to use pesticides and adopt new technologies since they can critically analyse and make own choices and therefore tend to adopt new technologies (Enete and Igbokwe 2009; Caleb and Ramatu 2013). Farmers who have more years of farming experience adopt technologies which tend to increase productivity (Idrisa et al. 2012) and are therefore less likely to use pesticide and decrease frequency of application. Finally, extension agents as well as FBOs introduce new technologies other than pesticides to farmers which influence farmers to less likely adopt pesticides to control pest and disease on their farm (Anang and Amikuzuno 2015). On the other hand, they may introduce farmers to new types of pesticides and advice farmers to increase the frequency of pesticide application in other to control pest effectively (Tiamiyu et al. 2009; Omolehin et al. 2007).

**Table 1:** Description, measurements and A-priori expectation of variables

Variable	Measurement	A-priori expectation
Gender	1 if male, 0 otherwise	+
Age	Years	+
Educational level	1 = No formal education, 2 = Primary, 3 = JHS, 4 = SHS, 5 = Tertiary	−
Years of farming experience	Years	−
Membership of FBO	1 = yes, 0 = otherwise	+/−
Access to extension service	1 = member, 0 = otherwise	+/−
Availability of agrochemical shop	1 = available, 0 = otherwise	+
Access to credit	1 = yes, 0 = otherwise	+
Cocoa income	Ghana cedi	+

## Results and discussion

### Demographic characteristics of cocoa farmers

Table 2 presents the results of the demographic characteristics of cocoa farmers in the study area. The domination by male respondents among the farmers could be the result of males having greater access to farm land than females. It could also be due to the fact that cocoa farming is more labour-intensive. Therefore, women are not able to meet the needed effort to cultivate the crop. The minimum age of the cocoa farmers was 20 years, maximum age was 68 years and the mean age was 44 years. This is comparable to that of the national average (Danso-Abbeam et al. 2014). The mean age indicates good quality of labour in cocoa production. This would have positive effects on productivity since younger farmers are more energetic and tend to adopt new technologies. The result on education shows that literacy level in the study area is high, although, very few farmers had tertiary education. Married farmers dominating cocoa farming means that individuals who engage in cocoa farming are married. This result is consistent with Bammeke (2003) who states that individuals who undertake agricultural activities are married. The average number of years of farming experience in the study area was 22 years. This means that people engaged in cocoa production are experienced in the study area.

**Table 2:** Demographic characteristics of respondents *Source*: Field work, 2015

Variable	Description	Frequency	Percentage (%)
Gender	Male	198	82.5
	Female	42	17.5
Age	20–39	49	20.4
	40–59	145	60.4
	Above 60	46	19.2
Marital status	Single	198	17.5
	Married	42	82.5
Educational level	No formal education	36	15.0
	Primary/JHS	118	49.2
	Middle/SHS	81	33.8
	Tertiary	5	2.0
Years of farming experience in cocoa	5–10	14	5.8
	11–15	39	16.3
	16–20	43	17.9
	Above 20	144	60.0

### Types and sources of pesticides used by cocoa farmers

Majority (85 %) of the respondents indicated they depend on chemicals to control pests and diseases while 15 % of the farmers used other forms of pest control such as IPM and ICM. The cocoa farmers (85 %) who depended on chemicals to control pests and diseases, used both COCOBOD approved and recommended pesticides and pesticides that are not approved by Ghana COCOBOD. The use of unapproved pesticides by cocoa farmers in the study area was attributed to the fact that the Ghana COCOBOD approved and recommended pesticides for cocoa production are not for sale, hence, are not readily available in the market or input shops. In Ghana, the only way a cocoa farmer can have access to the Ghana COCOBOD approved and recommended pesticides for cocoa production is through the Ghanaian government free “cocoa mass spraying” exercise.

It was however interesting to note that some cocoa farmers who benefited and used the approved and recommended Ghana COCOBOD pesticides indicated that pesticides in the open market (unapproved pesticides) were more effective than the approved ones. Although cocoa farmers claim unapproved pesticides are more effective compared to the approved and recommended Ghana COCOBOD pesticides, research by the Ghana COCOBOD reveals that approved and recommended pesticides are not harmful to pollinator insects of cocoa, for example, midges (Forcipomyia spp.) (COCOBOD 2014). Also, unapproved pesticides used in the cocoa industry are not screened at the Cocoa Research Institute of Ghana, to ensure that they comply with EU, Japanese and other markets requirements for food safety, maximum residual level (MRL) limits and sanitary and phyto-sanitary standards before they are used on cocoa, which could lead to the rejection of cocoa beans exported to these markets when traces/residues of these chemicals are found in the beans (Ghana News 2013). There is therefore, the need to educate cocoa farmers on the harmful effects of these unapproved pesticides on cocoa production.

Among the Ghana COCOBOD approved and recommended pesticides, Confidor, Akatemaster, Nordox, Kocide, Actara, Champion, Funguran, Metalm and Ridomil were mostly used by farmers in the study area. Table 3 presents the list of approved and recommended insecticides and fungicides by Ghana COCOBOD for the management of cocoa insect pests and diseases in Ghana and their main characteristics (i.e. active ingredient, main use and hazardous class) according to the World Health Organization (WHO) classification (WHO 2005).

**Table 3:** Insecticides and fungicides approved by Ghana COCOBOD for use by cocoa farmers in Ghana *Source*: Extracted from Adjinah and Opoku (2010) and Afrane and Ntiamoah (2011)

Trade name	Active ingredient	Main use	Chemical hazardous class (WHO)
Cocostar	Bifenthrin + Pirimiphos-methyl	Insecticides	II
Carbamult	Promecarb	Insecticides	
Akatemaster	Bifenthrin	Insecticides	II
Confidor	Imidacloprid	Insecticides	II
Fungikill	Cupric hydroxide + Metalaxyl	Fungicides	III
Metalm	Cuprous oxide + Metalaxyl	Fungicides	III
Champion	Cuprous hydroxide	Fungicides	
Kocide	Cupric hydroxide	Fungicides	III
Nordox	Cuprous oxide	Fungicides	
Funguran	Cuprous hydroxide	Fungicides	
Actara	Thiamethoxam	Insecticides	III
Ridomil	Metalaxyl cuprous oxide	Fungicides	III

II = moderately hazardous; III = slightly hazardous; (WHO 2005)

The pesticides that are not approved by Ghana COCOBOD for cocoa production but were used by the cocoa farmers in the study area includes: Akatesuro, Argine, Buffalo-Super, Lamtox, Sunpyrifos, Sumitox, DDT, Dursban, Pyrethroids-Decis, Kombat, Consider, Okumakete, Lambda-M, Condifor, Thiodan, Super-gro, Sumico-200EC, Confidence, Actala and Controller-super. Table 4 presents the most commonly used unapproved pesticides by the cocoa farmers and their main characteristics (i.e. active ingredient, main used and hazardous class) according to the World Health Organization (WHO) classification (WHO 2005).

**Table 4:** Commonly used unapproved pesticides by cocoa farmers in the study area *Source*: Field work, 2015

Trade name	Active ingredient	Main use	Chemical hazardous class (WHO)
Sunpyrifos	Chlorpyrifos-Ethyl	Broad spectrum	II
Lamtox	Lambda-Cyhalothrin	Insecticide	II
Okumakete	Thiamethoxam	Insecticide	III
Pyrethroids-Decis	Deltamethrin	Insecticide	II
Fastrack	Alpha-Cypermethrin	Insecticide	II
Polythrine	Cypermethrin	Insecticide	II
Dursban	Chlorpyrifos	Broad spectrum	II
Super 10	Permethrin	Broad spectrum	II
Consider Supa	Imidacloprid	Broad spectrum	II
Kombat	Lambda-Cyhalothrin	Insecticide	II
Aceta-star	Methyl-thiophanate	Pesticide/fungicide	III
Topsin-M	Methyl-thiophanate	fungicide	III
Condifor	Imidacloprid	Insecticide	II
Thiodan	Endosulfan	Insecticide	II
Sumitox	Fenvalerate	Insecticide	II
Lambda-M	Lambda-Cyhalothrin	Insecticide	II
Akatesuro	Diazinon	Insecticide	II
Argine	Aldrin		I
DDT	DDT	Insecticide	I
Sumico 200 EC	Fenvalerate	Broad spectrum	II
Confidence	Chlopyrifos/Lamda-cyhalothrin	Insecticide	II
Buffalo-Super	Acetamiprid/Chlorfenvinphos	Broad spectrum	I
Controller-Super	Lambda-Cyhalothrin	Broad spectrum	II

I = extremely hazardous; II = moderately hazardous; III = slightly hazardous; (WHO 2005)

Majority (85.8 %) of cocoa farmers who used the unapproved pesticides purchased their pesticides from agro-chemical shops whiles the rest (14.2 %) obtained their pesticides from other cocoa farmers. The farmers’ choice of unapproved pesticides was based on its effectiveness in controlling pest and disease (43.1 %), availability in the market (25.5 %), affordability (18.1 %) and recommendations by fellow famers (13.2 %).

### Frequency of pesticide application by cocoa farmers using pesticides

The frequency of pesticide application by cocoa farmers using pesticides ranged from one to nine times per growing season with a mean frequency of application of five times per growing season. This exceeds the Ghana COCOBOD recommended frequency of pesticide application (i.e. four times per season) (Adu-Acheampong et al. 2007; Danso-Abbeam et al. 2014). Cocoa farmers in the study area were found to have in-depth knowledge of the Ghana COCOBOD recommended and approved pesticides for use in cocoa production than the recommended frequency of pesticide application. The lack of knowledge of the Ghana COCOBOD recommended frequency of pesticides application per growing season could result in farmers using chemicals improperly. This can increase the issue of chemical residues in soils, harvested cocoa beans, water sources near cocoa farms as well as pesticide resistance and pest resurgence (Antwi-Agyakwa et al. 2015).

Out of the 204 (85 %) cocoa farmers who used pesticides, 95 of them (46.6 %) indicated they apply pesticides more than four times in the year under review whilst 24.5 % applied four times, 14.7 % applied three times, 9.3 % applied two times and 4.9 % applied once. Cocoa farmers applied pesticides based on different reasons. It was interesting to note that majority (52.5 %) of cocoa farmers indicated that the presence of insect pest and disease on cocoa informed them on when to apply pesticides whiles 17.6 % did routine (calendar) application of pesticides to control insect pests and diseases on their cocoa. Furthermore, 14.7 % of cocoa farmers depended on agrochemical dealers, 9.8 % consulted extension officers and 5.4 % depended on fellow farmers to apply pesticides. This confirms the report by Padi et al. (2000) which states that few cocoa farmers used the recommended pesticides at the recommended dosage, time and frequency. Cocoa farmers did not follow the recommended frequency of pesticide application as a result of increased pest and insect infestation. Ntow et al. (2006) note that during the wet season, farmers increased frequency of pesticide application, because pests and diseases proliferate during this period and increased wash-off by rainfall necessitated further application of pesticides.

### Probit result on the factors influencing pesticide use among cocoa farmers

Table 5 presents the probit result on the factors influencing cocoa farmers’ decision to use pesticide. The result reveals that seven variables out of nine variables estimated were significant. The significant variables were gender, age, educational level, years of farming experience, access to extension service, availability of agrochemical shop and access to credit. The result showed that the Wald Chi square value of 76.15 was significant at 1 % with log pseudolikelihood value of −61.132.

**Table 5:** Probit result on the factors influencing pesticide use among cocoa farmers *Source*: Author’s computation (2015)

Variables	Coefficient	Robust Std. Err.	P value	Marginal effect
Gender	0.681	0.298	0.022**	0.046
Age	−0.097	0.022	0.000***	−0.004
Educational level	0.371	0.146	0.011**	0.014
Farming experience	0.115	0.016	0.000***	0.004
Membership of FBO	0.233	0.304	0.443	0.008
Access to extension	−0.824	0.387	0.033**	−0.021
Availability of agrochemical shop	−0.988	0.300	0.001***	−0.061
Access to credit	0.758	0.309	0.014**	0.044
Cocoa income	0.497	0.340	0.144	0.019
Constant	−2.551	3.518	0.468	–

Diagnostic statistic	Value
Log pseudo likelihood	−61.132
Wald chi^2^	76.15
Prob > chi^2^	0.000
Pseudo R^2^	0.397

*** and ** represent 1 and 5 % significance level respectively

Gender was found to be positive and statistically significant at 5 %. This conformed to the a-priori expectation. This indicates that male farmers are more likely to use pesticide. This could be due to the fact that female farmers have higher health risk when they come in contact with pesticides and other chemicals (Engel et al. 2005; Goldner et al. 2010). In the northern part of Ghana, women are advised not to engage in pesticide application. Therefore, male farmers take up the activities of pesticide application. Again, Matlon (1994) and Nkamleu and Adesina (2000) have shown that agricultural technologies are more likely to be adopted by men compared to women.

Age was statistically significant at 1 % and negatively influenced the use of pesticide among cocoa farmers. This did not conform to the a-priori expectation. The result indicates that as the age of a farmer increases by 1 year, the probability that a farmer would use pesticide decreases. The result contradicts the findings of Alavalapati et al. (1995) which assert that young farmers are more likely to adopt new technologies than older farmers. The result could be explained by the fact that older farmers might have experienced possible health effects over the years from the use of pesticides. It is noted that farmers who have experienced health related issues from pesticide use are more concerned about health effects of pesticides than those who have not experienced such problems (Lichtenberg and Zimmerman 1999; Hashemi et al. 2012).

Educational level of a farmer positively influenced pesticide use and was statistically significant at 5 %. This did not conform to the a-priori expectation which indicates that educational level of a farmer negatively influences pesticide use. However, the result on educational level is in line with the findings of Nkamleu and Adesina (2000). This could be explained by the fact that other alternative pest control methods or technologies may not be readily available, hence, educated farmers would have no option than to use pesticide. Anang and Amikuzuno (2015) assert that farmers use inputs which are readily available to them, in order to save time and money in search of alternatives.

The result revealed that years of farming experience was statistically significant at 1 % and had a positive relationship with pesticide usage. This means that a year increase in farming experience increase the probability of pesticide use by a farmer. It was expected that years of farming experience would have a negative influence on pesticide usage since farmers with more years of farming experience are expected to have better skills and access to new information about improved technologies (Yasin et al. 2003; Idrisa et al. 2012). However, the result revealed otherwise. Again, the result could be due to unavailability of alternative technologies in controlling insect pests, therefore, making farmers to use pesticide. The result is in line with the findings of Idris et al. (2013).

Access to extension service was statistically significant at 5 % and negatively influenced pesticide use. This conformed to the a-priori expectation which showed that access to extension service decrease the probability to use pesticide. The result is in line with the findings of Anang and Amikuzuno (2015) which assert that access to extension service influence farmers to less likely adopt pesticides to control pest and disease on their farms. This is because extension agents may introduce new technologies other than pesticides to farmers. Therefore extension service could be used as an effective tool for reducing pesticide use among farmers.

Availability of agrochemical shop decreases the probability of a farmer to use pesticide. This did not conform to the a-priori expectation which indicates that the use of pesticide by farmers is positively influenced by availability of agrochemical shop. Availability of agrochemical shop was negative and statistically significant at 1 %. The result is surprising because it was expected that availability of chemical shop will positively influence cocoa farmers’ use of pesticides (Anang and Amikuzuno 2015). However, the result could be due to the fact that although agrochemical shops may be available to cocoa farmers, the cost of pesticides does not warrant them to use pesticides. Idris et al. (2013) and Adejumo et al. (2014) revealed that cost of pesticides reduce pesticide use among farmers.

Access to credit was statistically significant at 5 % and had a positive relationship with pesticide use. This means that a farmer’s access to credit increases the probability of a farmer to use pesticide. The result was at par with the a-priori expectation and could be explained by the fact that farmers may tend to afford and purchase more pesticide when they have access to credit. This is because they would be able to purchase the chemicals regardless of the cost (Abu et al. 2011).

### Tobit result on the factors influencing frequency of pesticide application

Table 6 presents the Tobit regression result on the factors influencing frequency of pesticide application. The Tobit model was significant at 1 % level with log pseudolikelihood value of −398.647. The significant factors which influenced frequency of pesticide application are years of farming experience, educational level of a farmer, access to credit, access to extension service, membership of farmer based organisation and cocoa income.

**Table 6:** Tobit result on the frequency of pesticide application *Source*: Author’s computation (2015)

Variables	Coefficient	Robust Std. Err.	P value
Gender	0.458	0.605	0.450
Age	0.007	0.020	0.746
Educational level	0.661	0.169	0.000***
Farming experience	−0.050	0.012	0.000***
Membership of FBO	−0.657	0.277	0.019**
Access to extension service	−2.233	0.301	0.000***
Availability of Agrochemical shop	−0.457	0.305	0.136
Access to credit	−0.867	0.430	0.045**
Cocoa income	−1.093	0.322	0.001***
Constant	14.880	3.935	0.000

Diagnostic statistic	Value
Log pseudolikelihood	−398.647
Prob > F	0.000
Pseudo R^2^	0.297

*** and ** represent 1 and 5 % significance level respectively

Years of farming experience was statistically significant at 1 % and had a negative relationship with frequency of pesticide application. This conformed to the a-priori expectation which shows that years of farming experience negatively influenced frequency of pesticide application. This means that as farming experience increase by one year, the frequency of pesticide application by a farmer reduces. Although years of farming experience positively influence use of pesticide among farmers, it reduced the frequency of pesticide application. This means that even though farmers purchase pesticide for use, they do not apply the pesticide indiscriminately or above the recommended frequency of pesticide application as this is likely to cause health and environmental hazards. According to Lichtenberg and Zimmerman (1999) and Hashemi et al. (2012), farmers who have experienced health problems from pesticide use are more concerned about health effects of pesticides.

Educational level of a farmer was statistically significant at 1 % and had a positive relationship with frequency of pesticide application. This did not follow the a-priori expectation which indicates that educational level of farmers would negatively influence frequency of pesticide application. The result is surprising but could be as a result of proliferation of insect pest and diseases on their cocoa farms. Ntow et al. (2006) revealed that farmers increased frequency of pesticide application because of proliferation of insect pests and diseases.

Membership of farmer based organisation was statistically significant at 5 % and negatively influenced frequency of pesticide application. This conformed to the a-priori expectation which indicates that FBOs negatively influenced frequency of pesticide application. The result means that farmers are becoming increasingly aware of insect pests thresholds as a result of being members of farmer based organizations. This indicates that farmer based organizations are reliable source of information to farmers.

Access to extension service was statistically significant at 1 % and negatively influenced frequency of pesticide application. This conformed to the a-priori expectation which indicates that access to extension service negatively influenced frequency of pesticide application. This means that as a farmer gains access to extension service, the frequency of pesticide application decreases. Hashemi et al. (2009) revealed that extension service is an effective method to promote rational use of pesticide.

There was a negative relationship between access to credit and frequency of pesticide application and this was statistically significant at 5 %. It was expected that access to credit would positively influence frequency of pesticide application, since cocoa farmers would have more purchasing power from the credit they obtain. However, the result revealed otherwise. This could be explained by the fact that although credit helps farmers to purchase more pesticides regardless of the price, they follow the recommended frequency of pesticide application, and hence, reduce the frequency of application or do not apply pesticide above the recommended frequency of application. This result contradicts the findings of Kebede et al. (1990) and Adesina (1996).

Cocoa income had a negative influence on frequency of pesticide application and was statistically significant at 1 %. This means that as a farmer’s income from sale of cocoa increases, the frequency of pesticide application reduces. The result is surprising, since it was expected that cocoa income would increase frequency of pesticide application. This contradicts the findings of Khan et al. (2015). The result indicates that cocoa farmers do not increase pesticide application as a result of higher income from sale of cocoa.

## Conclusion

Majority of cocoa farmers used pesticides (both approved and unapproved by Ghana COCOBOD) to control cocoa insect pests and diseases in the study area. Cocoa farmers’ decision to use pesticides in cocoa production was influenced by gender of a farmer, age of a farmer, educational level of a farmer, years of farming experience, access to extension service, availability of agrochemical shop and access to credit. In addition, the significant variables which influenced frequency of pesticide application were years of farming experience, educational level of a farmer, access to credit, access to extension service, membership of farmer based organisation and cocoa income.

## Recommendations

The Ghana COCOBOD should make approved and recommended pesticides readily available and affordable to cocoa farmers in the study area, since the use of unapproved pesticides was attributed to the unavailability of approved and recommended pesticides in agrochemical shops.

Access to extension service was one key factor which was found to reduce pesticide use and frequency of application among cocoa farmers in the study area. It is therefore recommended that extension service [provided by Ghana COCOBOD and the Ministry of Food and Agriculture (MoFA)] should target younger farmers as well as provide information on alternative pest control methods in order to reduce pesticide use among cocoa farmers. However, government policies such as the CODAPEC programme, also known as “cocoa mass spraying” exercise, which seeks to promote the use of pesticides among cocoa farmers should target male farmers and also provide subsidies on pesticides, as well as ensure cocoa farmers have access to flexible and affordable credit schemes. Again, extension service/agents should target cocoa farmers with less years of farming experience and encourage cocoa farmers to join farmer based organisations in order to decrease frequency of pesticide application.

Finally, since cocoa farmers’ use of pesticides and frequency of application was influenced by sources [agrochemical dealers, fellow farmers, presence of insect pest and disease and routine (calendar) application] other than extension service, the study recommends the intensification of extension education to cocoa farmers in the study area on pesticide use practices in order to avoid misuse and the risk factors associated with indiscriminate use of pesticide.

## Limitations of the study

The study had the following limitations: (1) it was difficult obtaining the list of cocoa farmers in the study area, which made it difficult to know the total number of cocoa farmers to be sampled. (2) Since the Berekum Municipality is one of the major cocoa producing areas in Ghana, a higher number of cocoa farmers could have been sampled. However, due to financial constraint in undertaking the research, only 240 cocoa farmers were sampled for the study.

## Suggestion(s) for further research

Further research could focus on how access to credit affect pesticide use via adoption of IPM.

## Abbreviations

GDP: gross domestic product
IPM: integrated pest management
IPNS: integrated plant nutrition systems
COCOBOD: Ghana Cocoa Board
FBO: farmer based organization
ICM: integrated crop management
DDT: dichlorodiphenyltrichloroethane
JHS: junior high school
SHS: senior high school
